# Influence of Accelerated Carbonation on the Physico-Mechanical Properties of Natural Fiber-Reinforced Lime Mortars

**DOI:** 10.3390/ma17184461

**Published:** 2024-09-11

**Authors:** Fotini Kesikidou, Ioanna Matamadiotou, Maria Stefanidou

**Affiliations:** Laboratory of Building Materials, Aristotle University of Thessaloniki, 54635 Thessaloniki, Greece; kesikidou@civil.auth.gr (F.K.); ioannmata@gmail.com (I.M.)

**Keywords:** accelerated carbonation, hot lime, air lime, hemp, lavender, mechanical properties

## Abstract

Lime mortars are considered the most compatible material for monuments and historic buildings, and they are widely used in restoration works. A key factor determining the mechanical and physical properties of lime mortars is carbonation, which provides strength and hardness. This paper indicates the properties gained in lime mortars produced by Ca(OH)_2_ and CaO reinforced with different bio-fibers (hemp and lavender) when exposed to the natural environment and in accelerated carbonation. At 90 and 180 days of manufacture, the mechanical and physical properties of the produced composites have been tested. The results show that the carbonation reaction works faster in the case of hot lime mortars, increasing their compressive strength by up to 3.5 times. Hemp-reinforced mortars led to an enhancement in strength by up to 30%, highlighting the significance of bio-fibers in facilitating CO_2_ diffusion. This was also verified by the thermogravimetric analysis and the determination of the carbon content of the samples. Optimal mechanical properties were observed in mixtures containing quicklime and hemp fibers when conditioned with 3% CO_2_ at the tested ages.

## 1. Introduction

Historically, lime was one of the most used building materials combined with others like clay, pozzolan, charcoal, gravel, straw, etc., depending on their role in construction (structural, rendering, and floor substrate) [1]. Nowadays, lime is mostly used for the restoration of historic buildings and monuments [2]. However, the current need to develop new sustainable building materials has brought lime binders to the foreground as the carbonation process and its ability to absorb CO_2_ can contribute to the prevention of climate change [3].

Traditionally, lime was used in pure air lime mortars either in the form of putty (Ca(OH)_2_) or by hot lime technology using CaO. Lime putty was made by the hydration of quicklime with a large amount of water, whereas in hot lime mortars, quicklime was used, in a gravel form, directly in the mixture along with the other wet ingredients, such as sand. Today, air lime is added in the form of powder in the mortars, which is the result of slaking with water as it is easier to handle and has stable consistency [4]. A review on hot lime mortars by RILEM TC 277-LHS summarizes the different properties compared to air lime mortars made with Ca(OH)_2_. The committee outlines that hot lime mixes stiffen faster due to the heat release and have a lower risk of slumping and leaching because of the expansion of quicklime and lower shrinkage. The microstructure of the mortars benefits from the released heat, which causes pores to interconnect, allowing air to enter and increase their frost resistance. Moreover, it seems that the slaking heat strengthens the adhesion of the binders with the aggregates and the cohesion of the mortar with the substrates [5,6].

Lime mortars harden by carbonation when exposed to air conditions. During the process, calcium hydroxide (Ca(OH)_2_) in the aqueous medium reacts with carbon dioxide (CO_2_) in the atmosphere, resulting in the formation of calcium carbonate (CaCO_3_) [7]. Carbonation has a high impact on many properties of the lime mortars. The change from portlandite (Ca(OH)_2_) to calcite (CaCO_3_) results in increased mechanical strength (flexural and compressive). The process seems to also affect the pore structure of the materials, reducing their total porosity. Eventually, the change in porosity influences the durability of the mortars [8].

On the other hand, several properties of the lime mortars seem to affect the carbonation like water/binder ratio, binder/aggregate ratio, the type of lime and aggregates, the use of additives (e.g., superplasticizers, air entrainers, fibers, etc.), CO_2_ concentration and the relative humidity in the atmosphere [7,8,9]. Silva et al. studied the performance of lime-based materials under different CO_2_ levels (0.05% and 5%). The authors investigated the properties of mortars with different water/binder ratios, mixing time, and superplasticizers. The results of the experiments indicated that accelerated carbonation at 5% occurred after 3 days of curing, while at 0.05%, the carbonation process was 40 times slower. Higher water amounts enhanced the carbonation at 5% but did not affect it at 0.05%. Longer mixing times of the mortars did not lead to higher carbonation rates, while the addition of superplasticizer hindered the process due to the reduction of porosity [7]. Rakhsh Mahpour et al. found that the addition of flax fibers affected the carbonation process differently at different humidity levels. It was stated that at 100% moisture conditions, the fibers enhanced the water and CO_2_ diffusion in the mortar matrix. On the contrary, at 0% humidity, the fibers seem to absorb any available water and allow carbonation only through direct absorption [10].

Fibers are used in mortars and concrete to improve their shrinkage behavior, tensile strength, toughness, and durability [11,12]. Although synthetic fibers such as steel, polypropylene, glass, nanofibers etc., are mostly used in construction, their high cost and carbon footprint have led many researchers to study their replacement with natural fibers [13]. Coconut [14], jute [15], kelp [16], bamboo [17], straw [18], and hemp [19] are some of the different types of natural fibers that have been investigated. The latter is one of the fastest-growing plants globally, absorbing high amounts of CO_2_ during its cultivation [20]. In terms of strength, the use of hemp seems to improve the behavior of the composites under flexure and compression [21].

The performance of natural fibers depends on their type, length, roughness, age, harvest method, and ability to absorb water [22]. Generally, their mechanical performance is acceptable, but lower than that of synthetic fibers. However, the interface between fibers and the matrix is another factor affecting their behavior in the composites. According to Azevedo et al., achieving a good fiber–matrix interface can improve the mechanical performance of the material, and this can occur by mechanical engagement due to the roughness on the surface of the reinforcement, electrostatic attraction, formation of chemical bonds or interdiffusion of materials [23]. Moreover, their chemical consistency also interferes with the matrix. Mwaikambo and Ansell [24] mentioned that the consistency of the fibers in cellulose and lignin has an impact on the hydration process and eventually on strength development. A previous work by the authors [25] found that the mechanical properties of jute-, coconut-, and kelp-reinforced cement and lime mortars showed a difference in the cooperation of the fibers depending on the type of binder. Cellulose-rich fibers such as jute and kelp worked better in lime mortars, while the properties of the cement mortars were improved when lignin-rich fibers such as coconut were used. In [26], lavender fibers were utilized as additives in 1.5% by volume in lime mortars and thus improved the flexure and thermal behavior (lower thermal conductivity). Pizzol et al. mentioned that the high pH level of Portland cement hinders the performance of vegetable/cellulose fibers, and thus accelerated carbonation can improve the interface between the fibers and the matrix through the reduction of pH [27].

This work intends to combine the above existing knowledge of the three different techniques: hot lime technique, carbonation mechanism and fiber reinforcement. For this purpose, quicklime and air lime in powder form were used for the production of mortars, with the addition of 1% (by volume) of hemp or lavender fibers. Quicklime was chosen based on a literature review of the mechanical performance of hot lime mortars, whereas air lime was chosen as a local material. Following the same pattern, hemp fibers were used based on their benefits, while lavender fibers were chosen as a local industrial by-product. The specimens were kept at different CO_2_ environments—0.05% and 3%—with relative humidity at 60%. The mechanical (flexural and compressive strength, ultrasound pulse velocity), and physical (porosity, capillary absorption, and carbonation) properties of the mortars were studied at 90 and 180 days of age. The aim is to study the influence of the carbonation conditions and the type of binder and reinforcement in the physico-mechanical properties of the mortars.

## 2. Materials and Methods

### 2.1. Materials and Mortar Composition

The plan for the experimental part was based on producing different types of lime mortars using air lime in powder form and hot lime technology (CaO) while the samples were reinforced with two types of bio-fibers. After production, the mortars were cured in a climatic chamber for 28 days and then placed at different CO_2_ conditions until testing at 90 and 180 days. Physical properties (porosity, capillarity, and carbonation depth), mechanical properties (flexure and compression) as well as microstructure of the exposed samples have been recorded and commented. The carbon and calcium carbonate contents of the samples after the age of 90 days were also determined.

**Figure 1:**
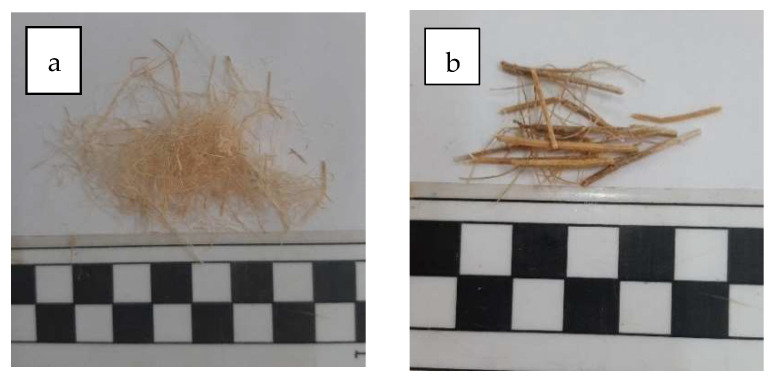

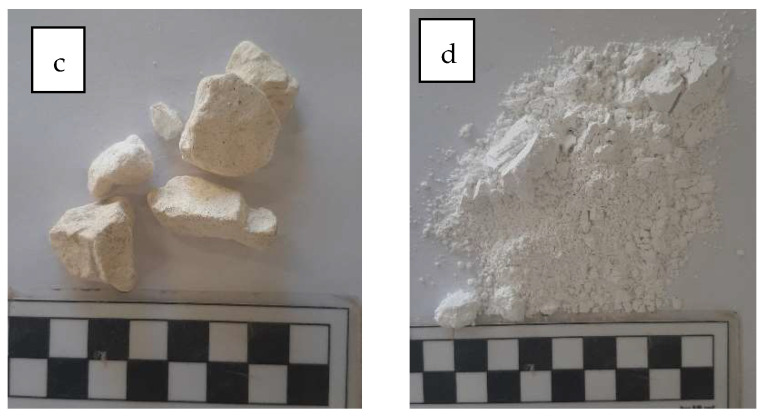
Hemp (**a**) and lavender (**b**) fibers, (**c**) quicklime, and (**d**) air lime.

**Figure 2:**
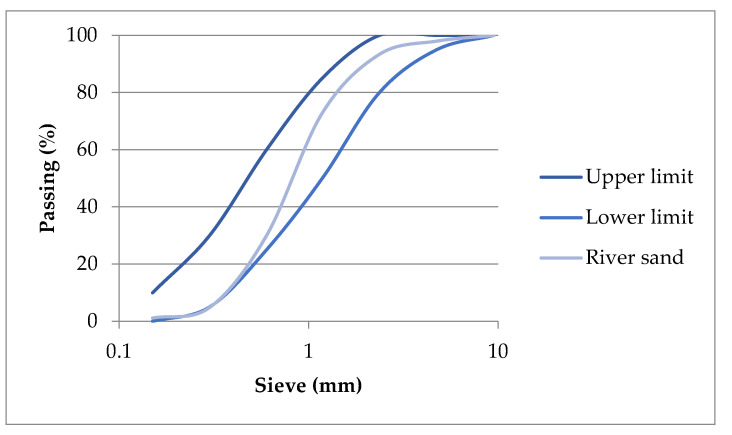
Granulometric curve of river sand (0–4) mm.

In particular, six lime mortar compositions were designed. A series of hot lime using CaO and powdered air lime using Ca(OH)_2_ mixtures were produced. These compositions were produced unreinforced and reinforced with 1% (by volume of the mortar) of hemp or lavender fibers. Based on the analysis of old mortars containing fibers [24], both of the fibers had 2 cm length (Figure 1). Quicklime, powder air lime CL90, and river sand (0–4) mm were the primary constituents of the produced mortars. The properties of the raw materials are given in Table 1. The granulometric curve of the river sand is shown in Figure 2.

For the production of the mortars, all ingredients were measured by volume. The compositions of the produced mortars are given in Table 2. Different mixing protocols were followed for the hot and powdered air lime mixtures.

For all mixtures, the binder/aggregate ratio was 1:3 by volume as a standard mortar composition [28]. For the hot lime mixtures, one part of quicklime and three parts of river sand (0–4) mm were mixed with one part of water and left until the exothermic reaction of lime slaking was completed. Afterward, one extra part of water was added and mixed to achieve the appropriate workability of the mortar as recorded by the flow table test [EN 1015-3] [29]. In the reinforced mixtures, the fibers (hemp or lavender) were added at the end of the mixing process. All fibers were used when saturated and surface dry to avoid any damage caused by the elevated temperatures during mixing. For the powdered air lime mortars, one part of hydrated lime CL90 was mixed with three parts of river sand (0–4) mm and one part of water. The fibers were added dry at the end of the mixing procedure. Keeping in mind their homogeneous distribution, i.e., water/binder ratio, was kept the same for hot lime and air lime mixtures, regardless of the effect on the workability of the mortars, to be able to compare the mechanical performance of the materials. Afterward, the material was placed in prismatic and cubic molds until hardened.

### 2.2. Curing Conditions

After demolding, all specimens were kept in a climate chamber with stable conditions (21 °C and 60%RH) for 28 days. At that age, the samples were dried in an oven at 60 °C for 24 h to reduce excess humidity, which could be a drawback of the carbonation process. Then, the samples were placed at different CO_2_ concentrations until testing at 90 and 180 days of the production period. Half of the produced mortars were kept at natural CO_2_ levels (0.05), and half of them in a 3% CO_2_ environment. The temperature and relative humidity at both chambers were kept stable (21 °C and 60%RH). The carbonation conditions were chosen to follow EN 12390-12 (Testing Hardened Concrete—Determination of the Carbonation Resistance of Concrete—Accelerated Carbonation Method) [30] and are summarized in Table 3. The letter “x” is used as an indicator for the mixtures that were kept at 0.05% CO_2_ environment.

### 2.3. Methods

Three prismatic specimens (4 × 4 × 16) cm were measured under flexure according to EN 1015-11 [31] at the ages of 90 and 180 days. After breaking, the halved prismatic specimens were used to record the compressive strength of the mortars according to EN 1015-11 [28].

A sonometer was used to determine the homogeneity of the prismatic samples at 90 and 180 days by measuring the ultrasound pulse velocity according to BS 1881-203 [32]. The equipment records the transit time of ultrasonic pulses through the specimen, which depends on the density and the elastic properties of the material.

The porosity of the mortars was measured according to the RILEM CPC 11.3 method [30]. Half-prisms, obtained after the flexure test, were dried at 60 °C in a ventilated oven and then kept fully immersed in water and under vacuum. After 24 h, the specimens were weighed to determine their porosity.

The capillary absorption of the lime mortars was determined according to EN 1015-18 [33] at 180 days of age. Prisms (4 × 4 × 16) cm were dried at 60 °C for 24 h in a ventilated oven and then placed in a tray and immersed in water to a depth of 2 mm. The weight of the specimens was recorded at different time intervals.

Cubic specimens (7 × 7 × 7) cm were kept at different CO_2_ environments to test the carbonation rate of the lime mortars at 90 and 180 days. The carbonation rate of the mortars was measured qualitatively by spraying a phenolphthalein solution on specimens broken in half. Phenolphthalein works as a pH indicator by changing color. When the pH is over 8.3, the solution turns pink, while in pH levels under 8.3, it remains colorless, and the material can be considered carbonated.

The carbon content of the samples—CaO, H and CaOx, Hx—was found using Dumatherm CN (Gerhardt, Königswinter, Germany) at 90 days. The steps of the analysis follow the Dumas method, including incineration of the sample with oxygen within the vertical combustion reactor, reduction of the nitrogen oxides, separation of the water, adsorption of CO_2_ and measurement of the nitrogen content in the gas resulting from combustion using a thermal conductivity detector, and desorption of CO_2_ and measurement of the carbon content using the thermal conductivity detector.

The carbonation of the CaO and H mixtures was also determined via thermogravimetric analysis (TG-DSC) performed with a STA449 F5 Jupiter NETZSCH (NETZSCH Analyzing & Testing, Mumbai, India). The amount of calcium hydroxide (Ca(OH)_2_) and calcium carbonate (CaCO_3_) in the samples were identified at the age of 90 days.

Microstructure analysis was performed by digital microscope (Dino-Lite, Torrance, CA, USA) at the age of 180 days in all specimens in order to record the presence of cracks and the adhesion of the fibers to the matrix. 

## 3. Results

**Figure 3:**
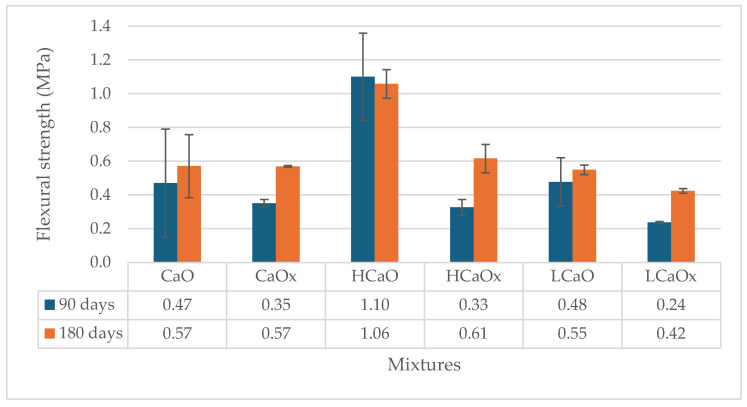
Flexural strength of hot lime mortars at 90 and 180 days.

**Figure 4:**
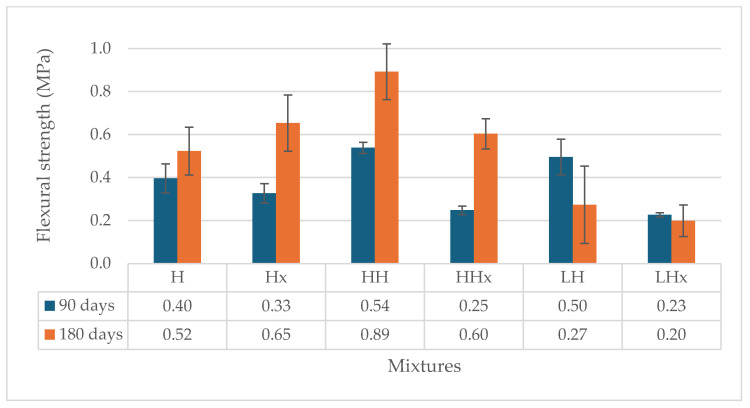
Flexural strength of powdered air lime mortars at 90 and 180 days.

The workability of the produced mixtures is given in Table 4. The addition of fibers (hemp or lavender) reduced the workability of the mortars. Additionally, the nature and the morphology of the fibers also affected workability. Moreover, the hot lime mixtures were less workable, indicating that more water was needed to achieve the same results as the powdered air lime mortars. This can be attributed to the heat released during the slaking process. The flexural and compressive strength of the reference mixtures CaO and H were determined at the age of 28 days. The results of the flexural strength of the lime mortars and the standard deviation of the measurements are given in Figure 3 and Figure 4.

The results of all mechanical properties and standard deviations (STDEV) of the measurements are listed in Table 5.

Comparisons were made regarding the carbonation conditions (3% CO_2_ vs. 0.05% CO_2_), the type of reinforcement (1% hemp or lavender fibers) and the type of mortar (hot lime or powdered air lime).

Regarding the carbonation reaction, it seems that the flexure of all mixtures (hot and powdered air lime) presents an increase when conditioned at 3% CO_2_. The carbonated CaO mix reached 0.47 MPa at 90 days, in contrary to 0.35 MPa, which was achieved by the mixture CaOx at 0.05% CO_2_. Similarly, the air lime H mixture (0.40 MPa) showed an uptake of 20% when carbonated conditions were applied compared to mixture Hx (0.33 MPa) kept at 0.05% CO_2_. The mortars kept at 3% CO_2_ seem to reach higher strength levels at 90 days, but the increase rate is slower over time. At 180 days, CaOx and Hx, exposed at 0.05% CO_2_, show the same results in flexure with the CaO and H kept at 3% CO_2_.

Moreover, the addition of 1% hemp or lavender fibers seemed to improve the results of flexure when the mortars were kept at 3% CO_2_. Hemp-reinforced hot lime mortar (HCaO) kept at 3% CO_2_ had an average flexural strength of 1.10 MPa, which is three times higher than the HCaOx kept at 0.05% CO_2_ (0.33 MPa). Likewise, when lavender was used as a reinforcement, LCaO achieved 0.48 MPa—double the strength of LCaOx—0.24 MPa at 90 days. This is an indication of the role of the fibers in the diffusion of CO_2_ in the matrix.

In relation to the type of fiber, it seems that hemp fibers work better in terms of strength development with both hot lime and powdered air lime mortars. The addition of the lavender fibers, on the other hand, did not seem to improve the flexural strength of the mixtures. More specifically, the flexure of the lavender-reinforced mortars kept at accelerated carbonation conditions (LCaO and LH) remained at the same levels as the unreinforced mortars (CaO and H). Furthermore, the mortars placed at 0.05% CO_2_ (LCaOx and LHx) showed a reduction of flexure almost at 50% compared to CaO and H, dropping from 0.33–0.35 MPa to 0.23–024 MPa at 90 d and from 0.57–0.65 MPa to 0.20–0.42 MPa at 180 d. This could be due to the absorption of the mixture water by the lavender fibers, i.e., a result of inadequate plasticity [25].

Generally, based on the binder, the hot lime mortars (-CaO- mixtures) presented a better performance during flexure compared to the powdered air lime mortars (-H- mixtures). Overall, HCaO with quicklime and hemp fibers conditioned at 3% CO_2_ achieved the best results in terms of flexure at both 90 and 180 days with 1.10 MPa and 1.06 MPa, respectively.

**Figure 5:**
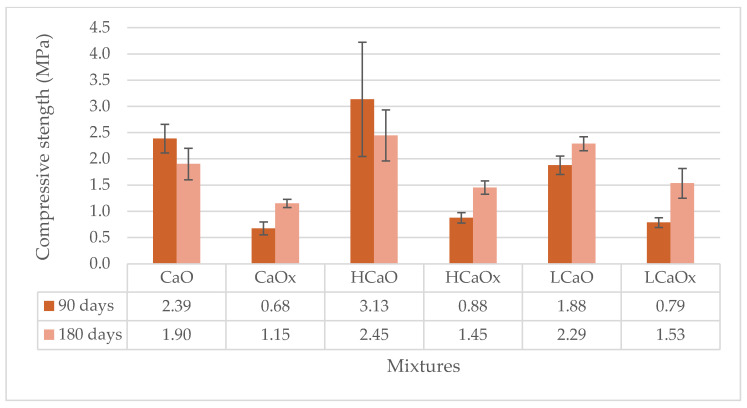
Compressive strength of the hot lime mortars at 90 and 180 days.

**Figure 6:**
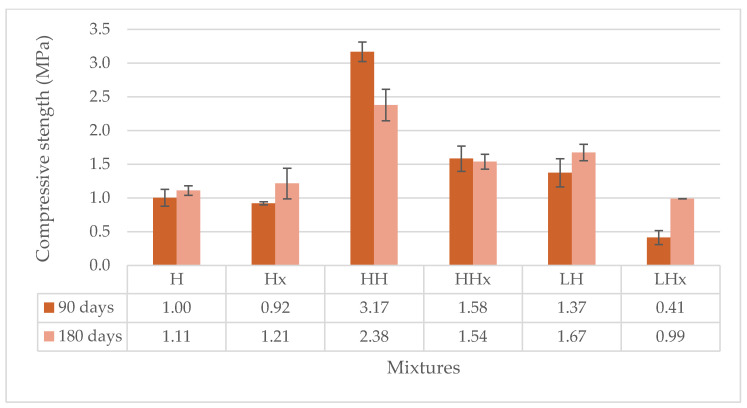
Compressive strength of the powdered air lime mortars at 90 and 180 days.

The significance of the accelerated carbonation conditions on the compressive strength of all mortars is clear in Figure 5 and Figure 6.

Hot lime mixture (CaO) kept at 3% CO_2_ had an increase in the compressive strength of 3.5-fold, reaching 2.39 MPa in relation to CaOx mixture conditioned at 0.05% CO_2_ at 0.68 MPa at 90 days. On the other hand, H and Hx air lime mixtures presented similar results in both conditions of approximately 0.92–1.00 MPa at 90 d and 1.11–1.21 MPa at 180 d age.

Again, the influence of the fibers on the carbonation mechanism is evident. Hemp- or lavender-reinforced hot lime mortars (HCaO and LCaO) achieved triple (3.13 MPa) or double (1.88 MPa) the results of HCaOx (0.88 MPa) and LCaOx (0.79 MPa), respectively. The addition of the fibers enhanced the diffusion of CO_2_ in the mortar, accelerating the carbonation mechanism and resulting in better compressive strength results.

However, in terms of the direct mechanical influence of the fibers, the hemp fibers improved the compressive strength of both the hot and powdered air lime mortars, but the results were slightly different when lavender was used. HCaO and HCaOx had an increase of 30%, reaching 3.13 MPa and 0.88 MPa, respectively, compared to the unreinforced mortars of CaO and CaOx with 2.39 MPa and 0.68 MPa, respectively. The compressive strength of powdered air lime mortars with hemp—HH, HHx—was 3.17 MPa and 1.58 MPa in comparison to H (1.00 MPa) and Hx mortars (0.92 MPa) at 90 days. A decrease was observed for HCaO and HH mixtures at the age of 180 days compared to 90 days. Nonetheless, both mixtures had higher compressive strength at 180 days when compared to the unreinforced CaO and H mixtures at the same age.

The influence of lavender fibers on the compressive strength is not clear, as reduction was observed in some cases. This is an indication of the poor adhesion of the fiber to the mortar matrix, which has been confirmed by optical observation of the samples with a microscope in Figure 12.

The behavior of the mortars under compression is different in relation to the type of mortar. For example, hot lime mortars present better results than powdered air lime mortars when conditioned at 3% CO_2_ for both 90 and 180 days. On the contrary, air lime mortars seem to record higher values under compression at 90 days when kept at 0.05% CO_2_. Eventually, at 180 days, both types of mortars (hot and powdered air lime) develop the same levels of strength. Based on this, it can be concluded that the accelerated carbonation mechanism works faster on hot lime mortars at early ages.

**Figure 7:**
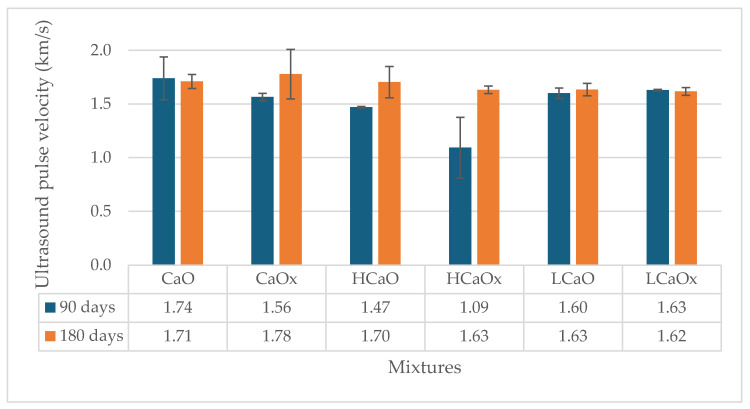
Ultrasound pulse velocity of the hot lime mortars at 90 and 180 days.

**Figure 8:**
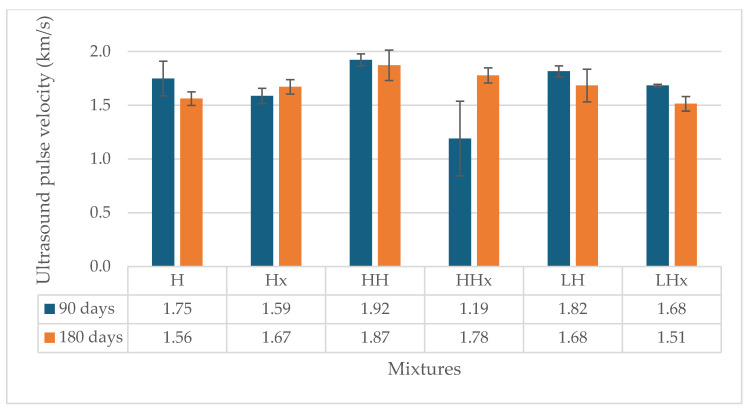
Ultrasound pulse velocity of the powdered air lime mortars at 90 and 180 days.

Figure 7 and Figure 8 present the results of the ultrasound pulse velocity test at 90 and 180 days. The standard deviation of the measurements is also given.

The mortars that have been subjected to accelerated carbonation have higher velocity values at 90 days, regardless of the type of mixture (hot or powdered air lime mortar), with the exception of LCaO. The increased results indicate a denser, more homogeneous matrix, which confirms the strength results. The highest uptake was observed for the hemp-reinforced hot and powdered air lime mortars. The results of the test are similar at 180 days of age.

**Figure 9:**
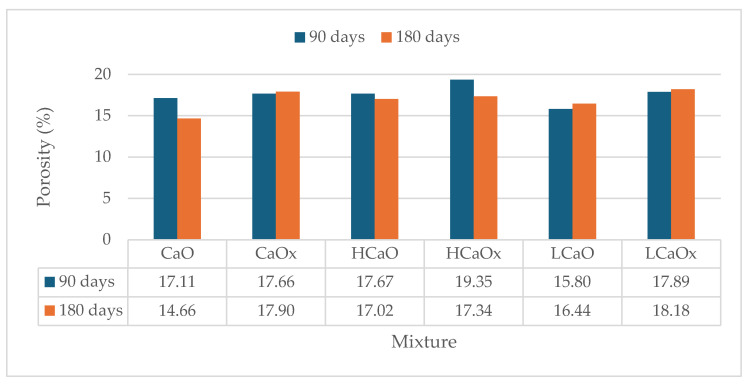
Porosity of the hot lime mortars at 90 and 180 days.

**Figure 10:**
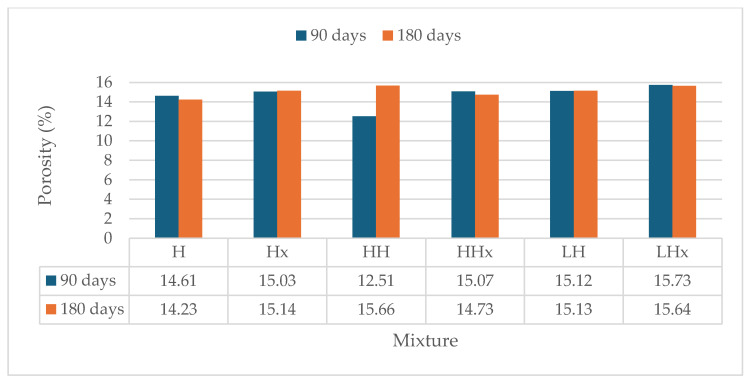
Porosity of the powdered air lime mortars at 90 and 180 days.

Open porosity values at 90 and 180 days are shown in Figure 9 and Figure 10. In general, mortars exposed at 3% CO_2_ appear to have slightly lower porosity values at 90 and 180 days with the reduction varying from 3% to 20%, with the exception of the hemp-reinforced mixture HHx at 180 days where porosity was reduced at 0.05% CO_2_.

The results correspond to the ultrasound pulse velocity test, resulting in the conclusion that these mortars have a denser structure that affects their mechanical performance.

Additionally, the porosity of the powdered air lime mortars decreased by 20% overall compared to hot lime mixtures. This could be attributed to the small calcitic lumps that formed in the latter mortars, which can potentially absorb water and increase the total porosity (detected with a microscope and shown in Figure 13).

Likewise, the addition of fibers led to a slight uptake of the porosity due to the ability of the fibers to absorb water. Hemp fibers appeared to have the largest increase of up to 10%. The morphology, length, and consistency of the fiber can affect the porosity of the samples.

The results of the capillary absorption coefficient at 180 days are given in Table 6. Hot lime mixtures conditioned at 3% CO_2_ present lower absorption rates, apart from the lavender-reinforced mixture, LCaO (4.501 kg/(m^2^·min^0.5^). However, with respect to the type of fiber, it seems that lavender fibers showed the lowest absorption rates among the hot lime mixtures (4.208 kg/(m^2^·min^0.5^). On the other hand, the addition of hemp increased the capillarity of the mortars (4.756 kg/(m^2^·min^0.5^). Capillary absorption of the air lime mortars had lower deviations. Overall, the capillarity rates of powdered air lime mixtures were lower that of the hot lime mixtures.

**Figure 11:**
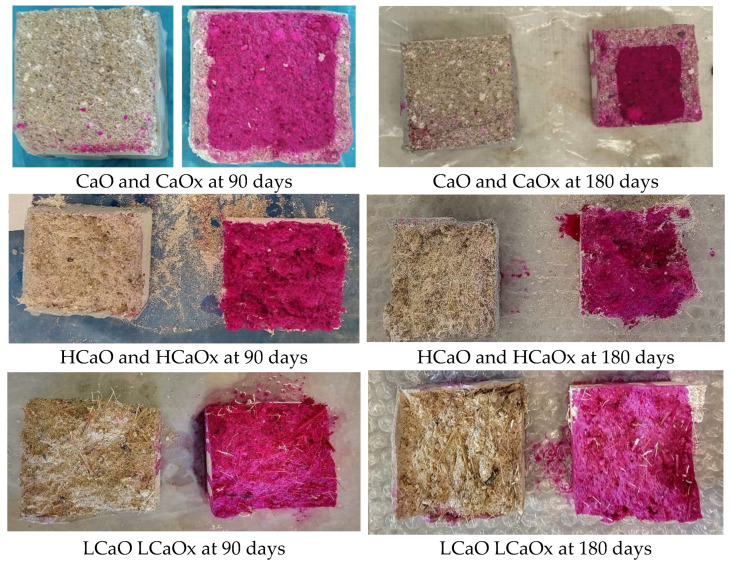
Phenolphthalein staining of hot lime mortars at 90 and 180 days.

**Figure 12:**
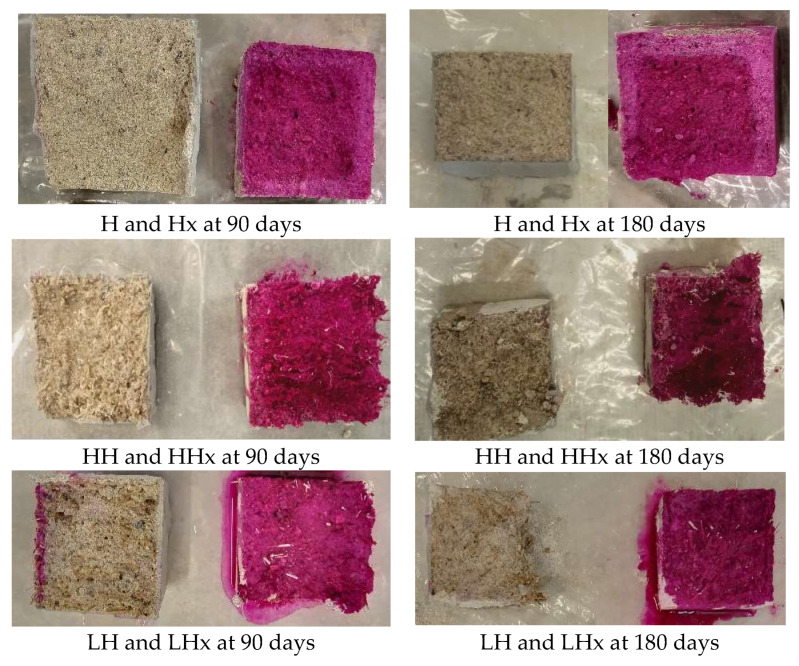
Phenolphthalein staining of air lime mortars at 90 and 180 days.

Based on the images taken after the phenolphthalein staining (Figure 11 and Figure 12), all mortars exposed to 3% CO_2_ were fully carbonated even from the age of 90 days if they were kept for at least 1 month in the CO_2_ chamber.

On the contrary, the solution had no or minor color differences for the mixtures conditioned at 0.05% CO_2_.

In relation to the fibers, it seems that the unreinforced mortars CaOx and Hx that were not subjected to accelerated carbonation have a slight alteration in color at their surface at 90 days, which gradually clears at 180 days. Also, it can be observed that the carbonation reaction of the hot lime mortars is more evident even for the specimens that were kept at 0.05% CO_2_ based on the clear color alteration at the perimetrical zone of the samples (~0.5 cm at 90 days and ~1 cm at 180 days).

The total carbon content of the unreinforced samples at the age of 90 days after exposure to 0.05% and 3% CO_2_ is given in Table 7. The results show an increase in the carbon content of the samples that were exposed to 3% CO_2_ as opposed to those that were kept at 0.05% CO_2_. The increase in the case of CaO mix was approximately 67%, whereas for the H mix, the uptake was 11%.

The calcium hydroxide and calcium carbonate content of the unreinforced samples that were kept at accelerated carbonation conditions (3% CO_2_) were identified by thermogravimetric analysis (Table 8). The analysis shows that the air lime mix (H), after exposure at 3% CO_2_, is fully carbonated, with total reaction of the calcium hydroxide. The formation of calcium carbonate is approximately 13.41%. On the other hand, in the quicklime mixture (CaO), the amount of calcium carbonate is higher (16.93%), with the remaining 5.38% of calcium hydroxide.

**Figure 13:**
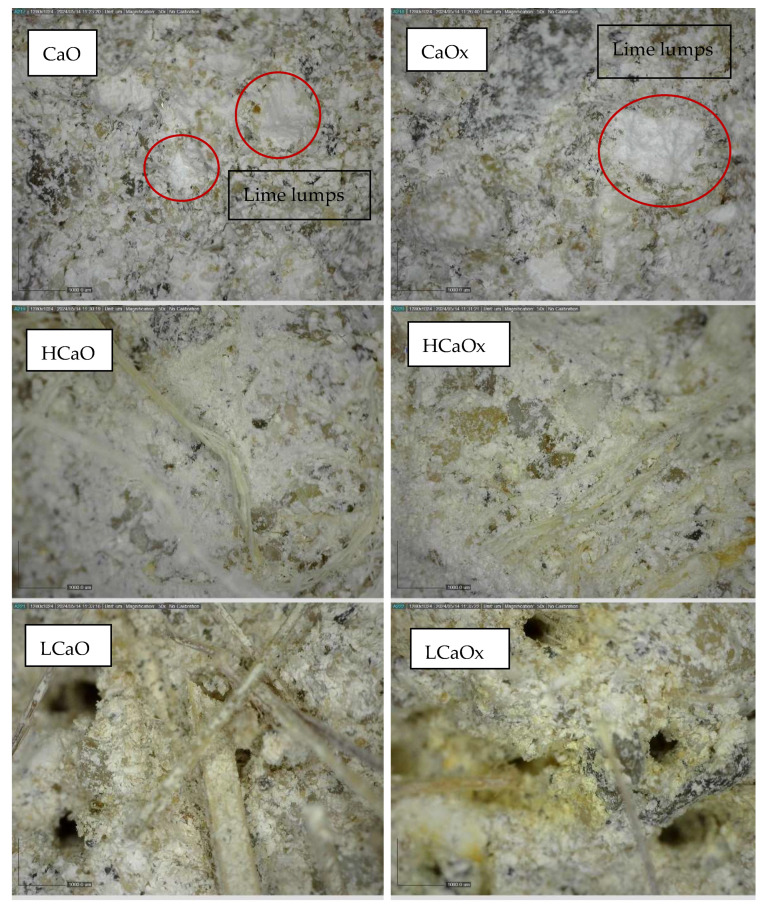

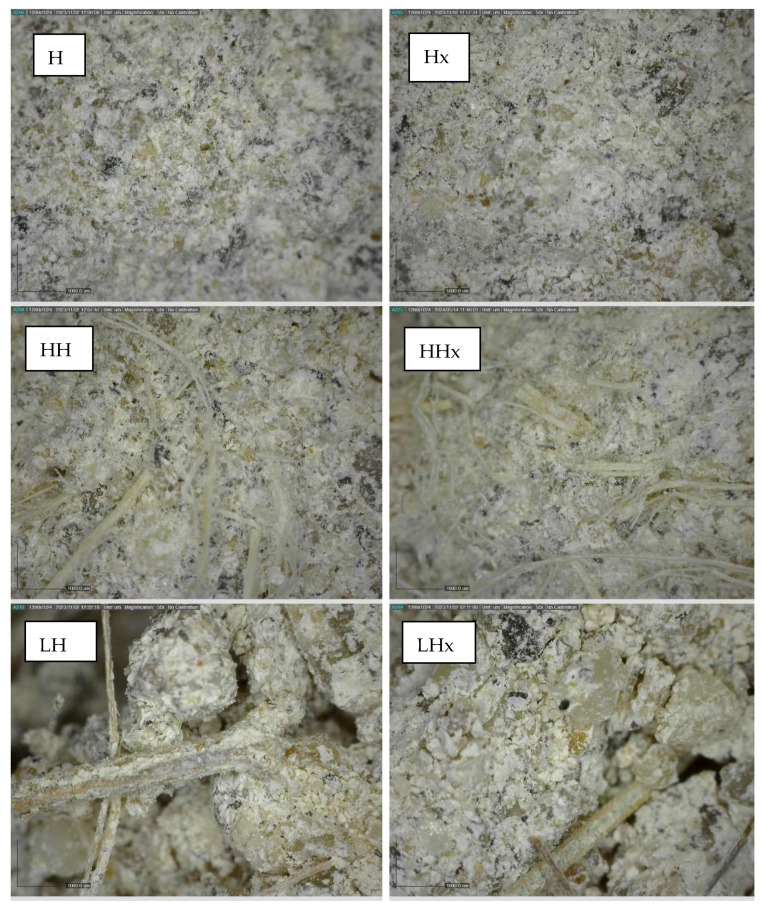
Microscopic images of the lime mortars at 180 days. scale bar = 1000 µm.

The microscopic observation of the samples (Figure 13) confirmed the above results. The scale of the figures, obtained by a digital microscope, is 1:1000 μm. In the case of hot lime mixtures, the mortars tend to have a homogeneous, dense structure. Small lime lumps, due to slaking, were detected in all samples, which is typical in this case [6].

Hemp fibers seem to have good dispersion and adhesion to the mortar matrix. On the other hand, although the lavender fibers are adequately dispersed in the matrix, large pores are detected, especially in the transition zone, which can explain the recorded mechanical properties. These pores are either created by the extraction of the fibers during flexure or by the poor fiber–binder interface. The thickness of the fiber could have also affected the mechanical performance of the mortars [34].

## 4. Discussion

Air lime mortars are known to gain strength at dry conditions by the reaction of calcium hydroxide (Ca(OH)_2_) in the aqueous medium with carbon dioxide (CO_2_) in the atmosphere, which results in the formation of calcium carbonate (CaCO_3_). This reaction mechanism has gained the interest of research, due to the ability of these mortars to absorb CO_2_. Several properties of the mortars can influence the rate of this reaction, such as the type of binder, the amount of water, the type and amount of the aggregates, the use of additives, etc. [8]. In this paper, the influence of the accelerated carbonation on the physico-mechanical properties of lime mortars produced under a different technology was studied in relation to the type of reinforcement (hemp or lavender fibers) and the type of binder (hot or powdered air lime).

Two series of air lime mortars were produced using different techniques. Quicklime was used to produce hot lime mixtures and hydrated lime (CL90) in powder form to produce air lime mortars. The mixtures were repeated with the addition of 1% by volume of hemp fibers and lavender fibers. Six mixtures were produced in total. After 28 days of curing in dry, stable conditions, the samples were split into two curing regimes. Half were kept at 3% CO_2_ and half at 0.05% CO_2_.

Based on the results, it seems that the exposure of all lime mortars to 3% CO_2_ enhanced the carbonation reaction. The mortars were fully carbonated at 90 days based on the phenolphthalein test. Anupama and Santhanam also observed that air lime mortars are fully carbonated at 14 days when exposed to 3% CO_2_ [35]. The carbonation had a clear effect on the mechanical performance of the hot lime mortars. In the case of H and Hx mixtures, the influence of the carbonation on the mechanical properties is not clear. However, it is evident when hemp or lavender fibers were added in the mixtures. Generally, all mixtures that were kept at 3% CO_2_ presented higher flexural and compressive strength, in some cases three times more than those kept at 0.05% CO_2_. The improvement in strength in higher CO_2_ environments was mentioned by other researchers. Ergenç and Fort tested the influence of high CO_2_ in lime mortars with different aggregates. The researchers concluded that the exposure of the mortars at 1600 ppm resulted in better mechanical and physical properties at early ages [36]. Oliveira et al. studied the effect of carbonation and humidity in air lime mortars. They found that the compressive strength rose over time with the increase of the depth of carbonation in the mortars [37].

Additionally, reinforcing the mortars with natural fibers seems to enhance the diffusion of CO_2_ resulting in higher compressive strength results. Lime mortars (hot and powdered air lime) with the addition of 1% hemp fibers showed improved mechanical characteristics. This is evident by the high increase in the compressive strength of the reinforced mortars exposed to 3% CO_2_ in comparison to those kept at 0.05% CO_2_. More specifically, the combination of hemp reinforcement and accelerated carbonation resulted in the best compressive strength values for the HCaO and HH mortars (3.13 MPa and 3.17 MPa, respectively). The HCaOx and HHx mixtures—exposed at 0.05%—reached 0.88 MPa and 1.58 MPa, respectively. Bui et al. also found that the addition of coconut fibers in cement increased the carbonation due to the air entrainment that allows CO_2_ diffusion [38]. On the other hand, the mortars with lavender fibers did not present high mechanical properties. The microscopic observation of the samples confirmed these results. The poor interface of the fiber with the matrix resulted in large pores in the mortar that reduced its mechanical capacity. This was also recorded in previous works [25].

In terms of the binder, it is noted that the carbonation mechanism seemed to work faster on the hot lime mixtures. This is noticed by the increased flexural and compressive strength of the mortars at 90 days, but also by the higher carbon and calcium carbonate content of the samples. An uptake of 67% in the carbon content was found in the CaO mixture, compared to 11% in the H mixture. Additionally, calcium carbonate was found 16.93% for CaO at 90 days versus 13.41% for H mix. As mentioned by Brown, hot mixed mortars have higher permeability [39], which could be responsible for the faster carbonation of the mortars.

Finally, the ultrasound pulse velocity values are slightly increased at 90 days for the carbonated mixtures, indicating a denser structure of the mortar. At 180 days, the results are similar. The porosity and capillary absorption also rose due to the addition of the fibers and the type of mortar.

## 5. Conclusions

This paper focuses on the investigation of the influence of carbonation on the phyico-mechanical properties of hot and powdered air lime mortars reinforced with hemp or lavender fibers.

In an effort to combine principles of compatibility with sustainability in lime-based mortars for restoration works, the research focuses on bio-fibers (instead of plastic or other energy-consuming materials). The comparison of the two different bio-fibers clearly indicated the beneficial role of hemp.

- A different technology of lime mortars is tested (hot-lime and powdered air lime) in an accelerated CO_2_ environment. The results of this research prove that the hot lime mixing technique and natural reinforcement can be combined with accelerated carbonation conditions to improve the physico-mechanical properties of the mortars. In conclusion, all the mortars were fully carbonated at 90 days when kept at 3% CO_2_.
- The exposure of the lime mortars to 3% CO_2_ greatly improved the strength development, especially at early ages.
- Hemp fibers enhanced the diffusion of CO_2_ in the mortars, thus resulting in better mechanical properties.
- Hemp fibers had a good dispersion and adhesion within the mortar structure. On the contrary, the lavender fibers cooperated poorly with the binder, resulting in lower strength results.
- The accelerated carbonation conditions seemed to work better in hot lime mixtures and improved the mechanical performance of the lime mortars more quickly. This was confirmed by the higher carbon and calcium carbonate content of the samples at 90 days.

Further investigation on the percentage of CO_2_ and the use of other types of local natural fibers (e.g., seaweed) can provide more evidence of the benefits of the accelerated carbonation mechanism on the properties of lime mortars.

## Figures and Tables

**Table 1:** Properties of the raw materials.

Material	Ca(OH)_2_ (%wt)	CaCO_3_ (%wt)	Thickness (mm)	Specific Gravity (g/mL)	Water Absorption (%)
Quicklime	4.48	0.86	-	3.231	-
Air lime	82.99	11.53	-	2.279	-
River Sand (0–4 mm) (siliceous)	-	-	-	2.655	-
Hemp	-	-	0.1–0.2	1.661	75
Lavender	-	-	0.3–0.6	1.273	78

**Table 2:** Compositions of the produced lime mortars (parts per volume).

Mixture	Quicklime	Air Lime	River Sand (0–4) mm	Water	Hemp Fibers (% by Vol.)	Lavender Fibers (% by Vol.)
CaO	1	-	3	2	-	-
HCaO	1		3	2	1	-
LCaO	1	-	3	2	-	1
H	-	1	3	1	-	-
HH	-	1	3	1	1	-
LH	-	1	3	1	-	1

**Table 3:** Carbonation conditions of the lime mortars.

Mixture	Chamber with Natural Carbonation (0.05% CO_2_), 21 °C, 60%RH	Chamber with Accelerated Carbonation (3% CO_2_), 21 °C, 60%RH
CaO, HCaO, LCaO, H, HH, LH		v
CaOx, HCaOx, LCaOx, Hx, HHx, LHx	v	

**Table 4:** Workability and 28 d strength of the lime mortars.

Mixture	Workability (cm)	Flexural Strength at 28 d (MPa)	Compressive Strength at 28 d (MPa)
CaO	13.5	0.32	1.06
HCaO	12.8	-	-
LCaO	12.0	-	-
H	16.2	0.29	0.80
HH	13.5	-	-
LH	14.7	-	-

**Table 5:** Mechanical properties of lime mortars at 90 and 180 days.

Mixture	Age (Days)	Flexural Strength (MPa)	STDEV	Compressive Strength (MPa)	STDEV	Ultrasound Pulse Velocity (km/s)	STDEV
CaO	90	0.47	0.32	2.39	0.27	1.74	0.20
CaOx	90	0.35	0.02	0.68	0.12	1.56	0.03
H	90	0.40	0.07	1.00	0.12	1.75	0.16
Hx	90	0.33	0.05	0.92	0.02	1.59	0.07
HCaO	90	1.10	0.26	3.13	1.09	1.47	0.01
HCaOx	90	0.33	0.05	0.88	0.10	1.09	0.28
HH	90	0.54	0.03	3.17	0.14	1.92	0.06
HHx	90	0.25	0.02	1.58	0.19	1.19	0.35
LCaO	90	0.48	0.14	1.88	0.18	1.60	0.05
LCaOx	90	0.24	0.01	0.79	0.09	1.63	0.01
LH	90	0.50	0.08	1.37	0.21	1.82	0.05
LHx	90	0.23	0.01	0.41	0.10	1.68	0.01
CaO	180	0.57	0.19	1.90	0.30	1.71	0.07
CaOx	180	0.57	0.01	1.15	0.08	1.78	0.23
H	180	0.52	0.11	1.11	0.07	1.56	0.06
Hx	180	0.65	0.13	1.21	0.23	1.67	0.07
HCaO	180	1.06	0.08	2.45	0.49	1.70	0.15
HCaOx	180	0.61	0.08	1.45	0.13	1.63	0.04
HH	180	0.89	0.13	2.38	0.23	1.87	0.14
HHx	180	0.60	0.07	1.54	0.11	1.78	0.07
LCaO	180	0.55	0.03	2.29	0.13	1.63	0.06
LCaOx	180	0.42	0.01	1.53	0.28	1.62	0.04
LH	180	0.27	0.18	1.67	0.12	1.68	0.15
LHx	180	0.20	0.07	0.99	0.00	1.51	0.07

**Table 6:** Capillary absorption coefficient of the lime mortars at 180 days.

Capillary Absorption Coefficient kg/(m^2^·min^0.5^)	3% CO_2_	0.05% CO_2_
CaO	4.061	4.609
HCaO	3.922	4.756
LCaO	4.501	4.208
H	3.838	4.151
HH	3.341	4.132
LH	3.787	4.101

**Table 7:** Carbon content after exposure to CO_2_ at the age of 90 days (DUMATHERM).

Sample	Carbon (%)
CaO	2.915
CaOx	1.740
H	1.432
Hx	1.290

**Table 8:** Carbonation of the samples at 90 days (TG/DSC) (% wt.).

Sample	Ca(OH)_2_	CaCO_3_
CaO	5.38	16.93
H	<0.01	13.41

## Data Availability

The original contributions presented in the study are included in the article, further inquiries can be directed to the corresponding author.
